# Supplementary figures and images for: Host-derived circular RNAs display proviral activities in Hepatitis C virus-infected cells
Source: PLoS Pathog. 2020 Aug 7;16(8):e1008346. doi: 10.1371/journal.ppat.1008346 (PMC7437927; doi:10.1371/journal.ppat.1008346)

S1 Fig

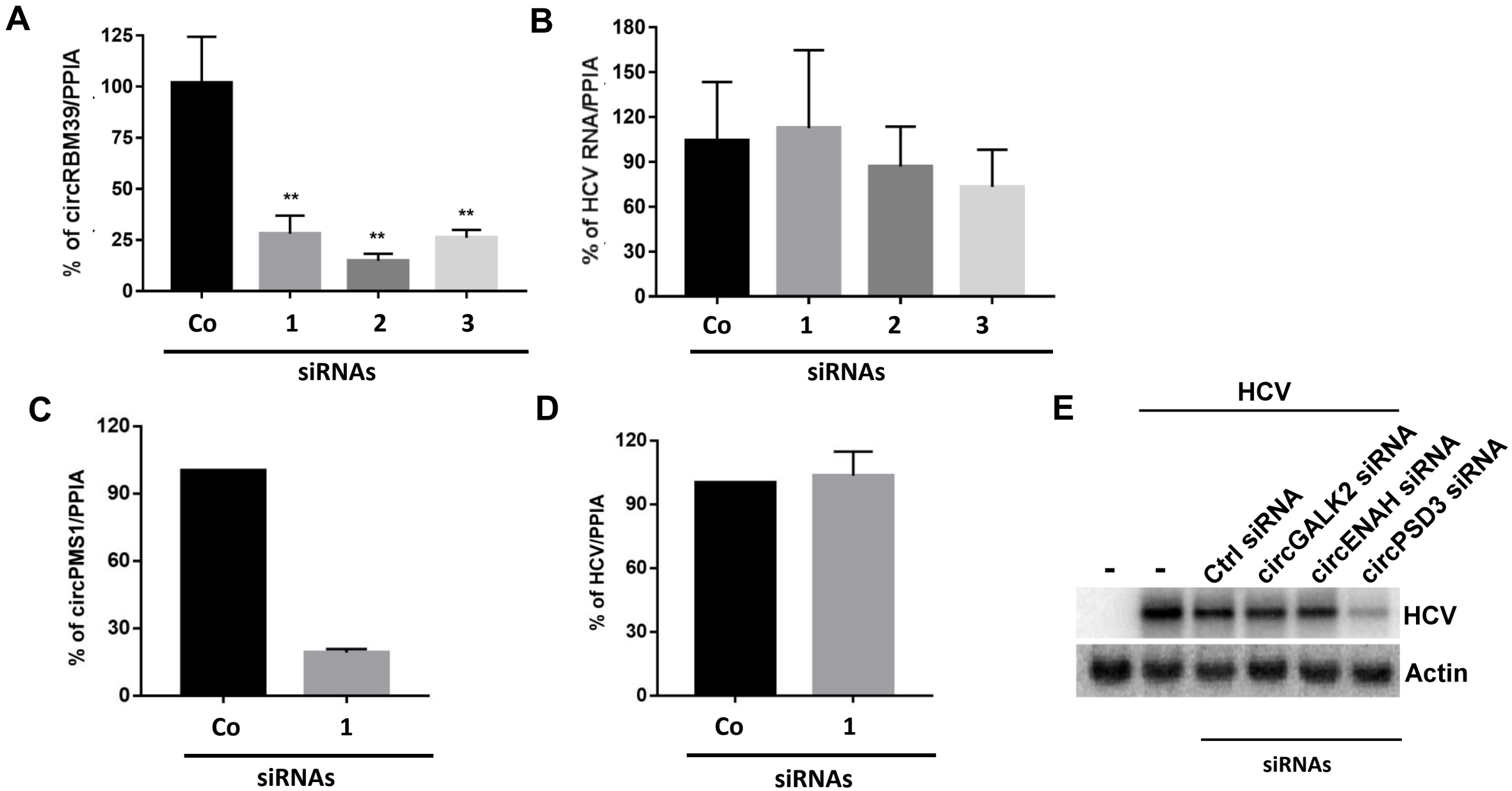

Supplement: S1 Fig — Effects of control and three siRNAs directed against circRMB39 (A,B) or circPMS1 RNA (C,D) on HCV RNA abundance. RNA abundance was determined by RT-qPCR. (E) Effects of various circRNA depletions on HCV RNA abundances, examined by Northern blot analyses. (PDF) [file ppat.1008346.s001.pdf]

S2 Fig

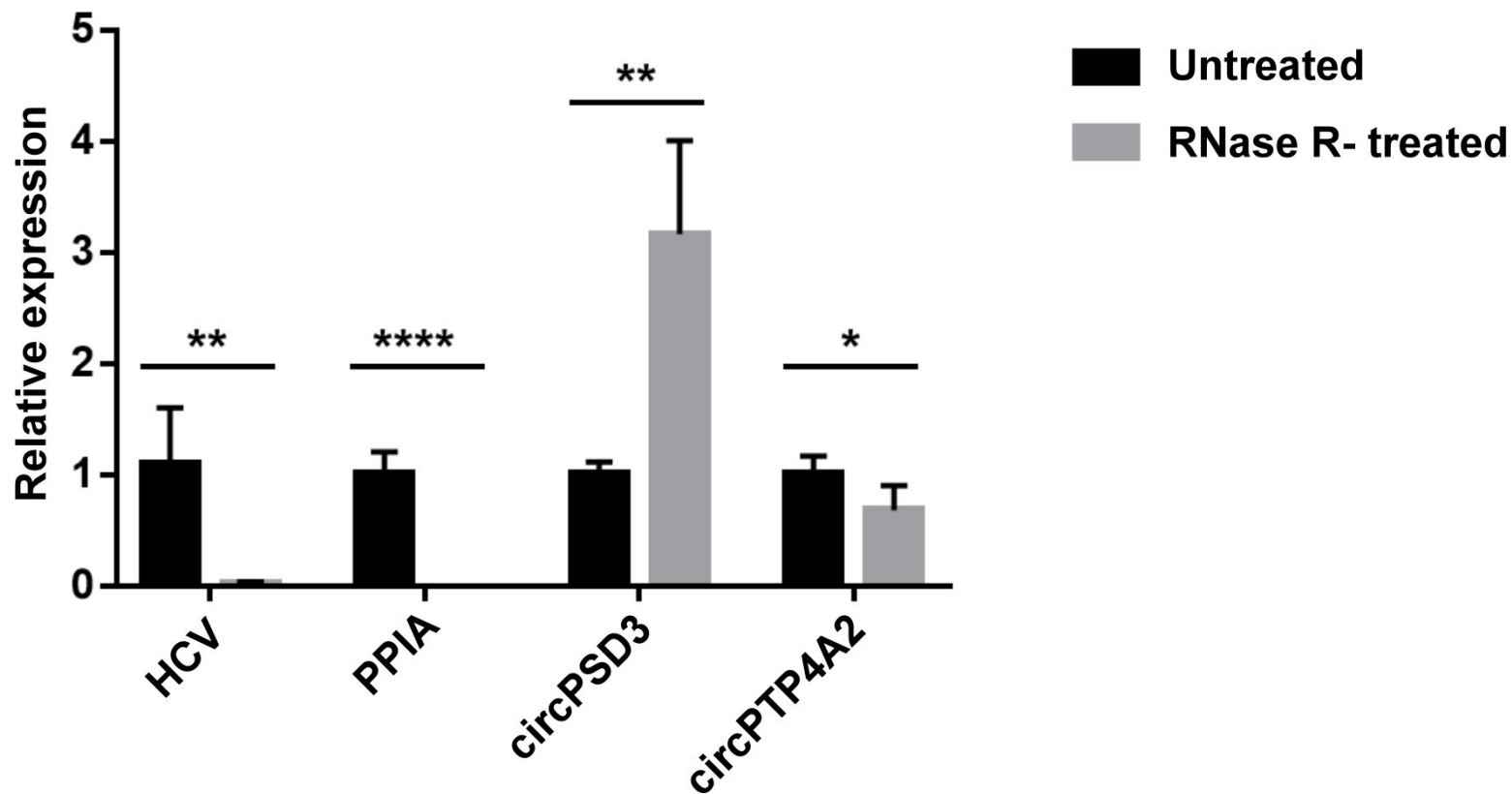

Supplement: S2 Fig — Total RNA from JFH1-infected cells was treated with or without RNase R. RNA abundances were analyzed using RT-qPCR. The RNA abundances are compared to RNA abundances from the untreated samples (set to 1.0). circPTP4A2 is derived from protein tyrosine phosphatase 4A2 mRNA. (PDF) [file ppat.1008346.s002.pdf]

S3 Fig

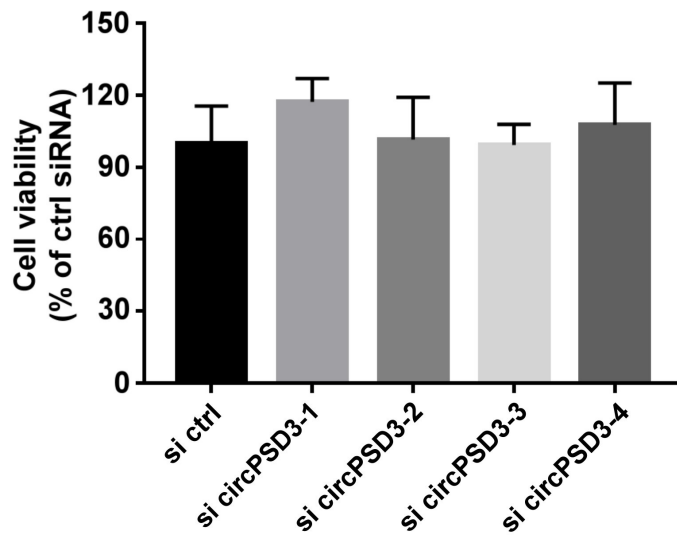

Supplement: S3 Fig — The cell viability of control siRNA and four circPSD3 siRNAs were measured at two days after infection. The data are representative of three independent experiments. (PDF) [file ppat.1008346.s003.pdf]

**S4 Fig**

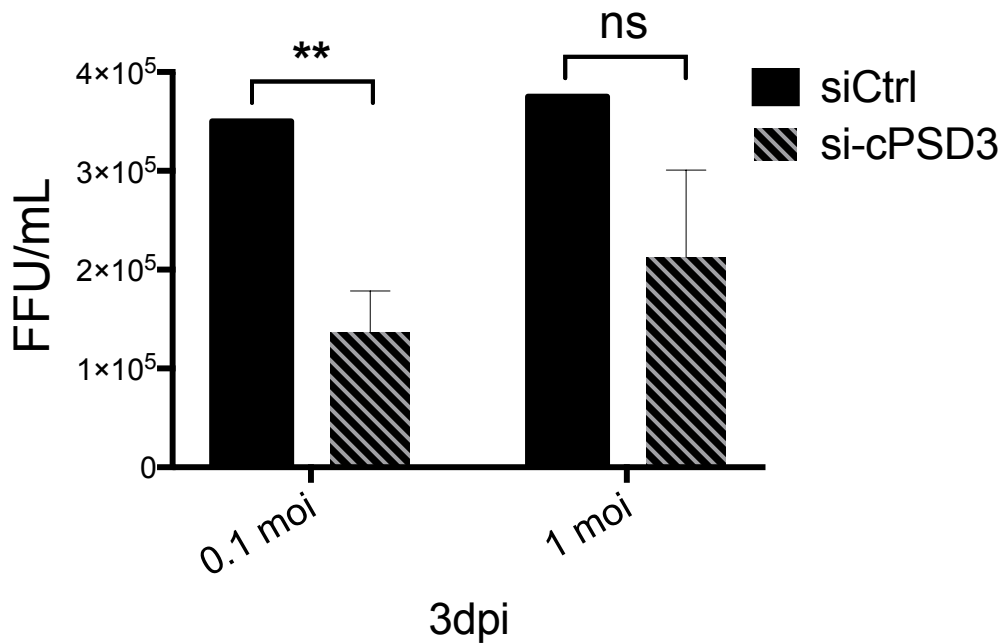

Supplement: S4 Fig — Huh7 cells were transfected with non-targeting control siRNAs (siCtrl) or siRNA targeting circPSD3 (si-circPSD3). At one day post transfection, cells were infected with JFH-1 virus at 0.1 moi or 1 moi. Supernatants were collected at three days post infection and viral titers were determined by focus forming assays (FFU). (PDF) [file ppat.1008346.s004.pdf]

S5 Fig

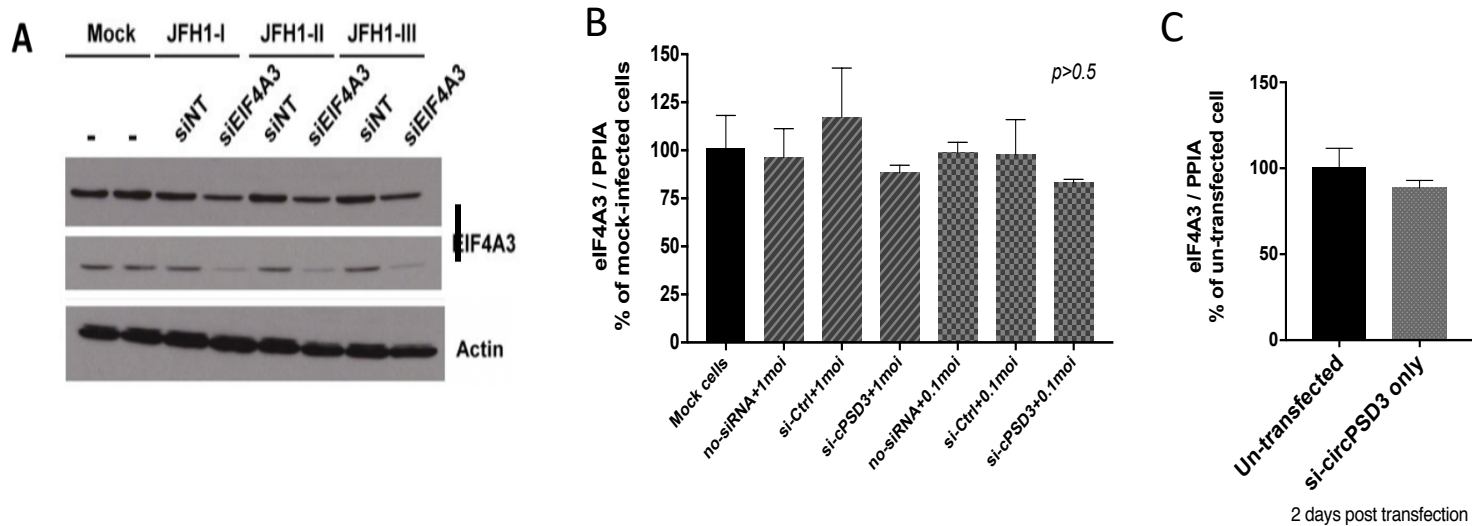

Supplement: S5 Fig — (A) eIF4A3 protein abundances were measured by Western blot at three days after HCV JFH-1 infection. Three independent experiments are shown. (B) eIF4A3 mRNA abundances in siRNA-transfected cells that were further infected with HCV. Mock cells are non-transfected and non-infected cells. Data from RT-qPCR reactions are shown. (C) Effects of circPSD3 depletion on eIF4A3 mRNA abundances in uninfected cells. Data from RT-PCR are shown. (PDF) [file ppat.1008346.s005.pdf]

S6 Fig

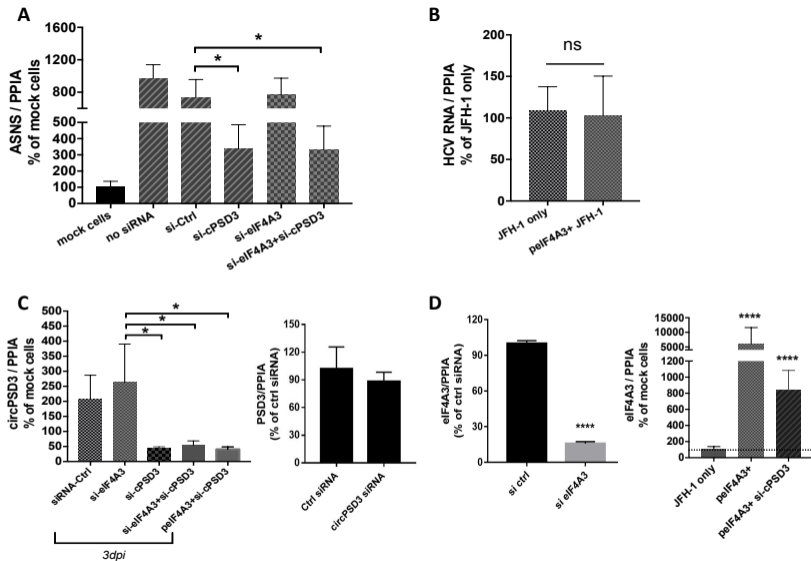

Supplement: S6 Fig — (A) Cells were transfected with siRNA targeting circPSD3 or eIF4A3, or co-transfected with both siRNAs. At one day post transfection, cells were infected with JFH-1 at 0.5 moi. ASNS abundances were measured 3 days post infection by RT-qPCR. (B) Cells were transfected with plasmid peIF4A3. At one day post transfection, cells were infected with JFH-1 at 0.5 moi and incubated for 3 days. HCV RNA abundances were measured by RT-PCR. (C) Knockdown efficiencies of individual siRNA transfections on circPSD3 and linear PSD3 RNA abundances. (D) eIF4A3 RNA abundances after transfection with siRNA or peIF4A3 plasmid. RNA abundances were evaluated by RT-qPCR after cells were transfected and further infected for 3 days. Data from three independent experiments are shown (* p<0.05; ****p<0.0001). (PDF) [file ppat.1008346.s006.pdf]
