# Supplementary material for: Host-derived circular RNAs display proviral activities in Hepatitis C virus-infected cells
Source: PLoS Pathog. 2020 Aug 7;16(8):e1008346. doi: 10.1371/journal.ppat.1008346 (PMC7437927; doi:10.1371/journal.ppat.1008346)
Supplement: S1 Table — The table shows the circRNAs used in this study, including gene name, circle name, sizes of circRNAs, linear RNAs, and primers (5’-3’) used for the qPCR-based validations. (PDF) [file ppat.1008346.s007.pdf]

S1 Table

Gene name	Circle name	CircRNA	Linear RNA	Primers
Exosome Component 3 (EXOSC)	hsa_circ_0005887	271 nts	1145 nts	circEXOSC FW: CGGAAAGTAGCCCGAGTACA<br>circEXOSC RV: TCACTTTGGCCAAGACAATG
TIA1 Cytotoxic Granule Associated RNA Binding Protein Like 1 (TIAL1)	hsa_circ_0004498	1259 nts	3945 nts	circTIAL1 FW: AATCACCTTCTGCTGCTTGG<br>circTIAL1 RV: ACCATCATTTTGGAGTTTGG
RNA Binding Motif Protein 39 (RBM39)	hsa_circ_0115058	4436 nts	5132 nts	circRBM39 FW: TGCCTCCTTGGAAAGCTCTA<br>circRBM39 RV: TTTGACTCCTTCGGCACTTT
Pleckstrin And Sec7 Domain Containing 3 (PSD3)	hsa_circ_0004458	448 nts	11704 nts	circPSD3 FW: CCATTGCTTCACAAGATGGA<br>circPSD3 RV: CCAGCATTGGTGGCCATGT
