# Supplementary material for: Host-derived circular RNAs display proviral activities in Hepatitis C virus-infected cells
Source: PLoS Pathog. 2020 Aug 7;16(8):e1008346. doi: 10.1371/journal.ppat.1008346 (PMC7437927; doi:10.1371/journal.ppat.1008346)
Supplement: Attachment — Submitted filename: response.to.reviewers.final.docx [file ppat.1008346.s008.docx]

**Responses to the reviewers’ comments**

We are grateful for the reviewers’ critiques which were very helpful in improving our manuscript on the roles for host-derived cirRNAs during HCV infection. We have further solidified that both circPSD3 and eIF4A3 play roles in HCV-infected cells. However, further experiments acknowledged that combined roles for circPSD3 and eIF4A3 in NMD in virus-infected cells and uninfected cells are difficult to decipher, mostly because viral core protein expression and its role in NMD may preclude a more detailed analysis in infected cells. Additionally, rescue experiments that involve over-expression of eIF4A3 have shown little effects on NMD, likely because they rely on the stoichiometric expression of eIF4A3/Y14/Magoh complexes. **However,** **our results strongly argue that both circPSD3 and eIF4A3 have distinct roles in modulating HCV RNA abundances.**

It is stated on line 38 in the Abstract: *One particular up-regulated circRNA, circPSD3, displayed a very pronounced effect on viral RNA abundances in both hepatitis C virus- and Dengue virus-infected cells. Though circPSD3 has been shown to bind factor eIF4A3 that modulates the cellular nonsense-mediated decay (NMD) pathway, circPSD3 regulates RNA amplification in a pro-viral manner at a post-translational step, while eIF4A3 exhibits the anti-viral property of the NMD pathway. Findings from the global analyses of the circular RNA landscape argue that pro-, and likely, anti-viral functions are executed by circRNAs that modulate viral gene expression as well as host pathways. Because of their long half-lives, circRNAs likely play hitherto unknown, important roles in viral pathogenesis.*

We appreciate the Editors patience during the long delay of submitting this revision because of COVID-19-induced lockdown of our research facilities in the US and Spain. We have added Dr. Qian Cao as an author, because she has collected most of the new data in the revised manuscript.

**Part II – Major Issues: Key Experiments Required for Acceptance**

**Reviewer #1:** No major issues

**Reviewer #2:** Major concern of the study that need to be addressed are:

1. How does HCV regulate eIF4A3 levels? Does circPSD3 play a role in negatively regulating eIF4A3.

Response: HCV infection does not alter the abundances of eIF4A3 mRNA and eIF4A3 protein. Furthermore, depletion of circPSD3 does not alter the abundance of eIF4A3 mRNA.

This is shown in **new Suppl. Fig. 5** and described on line 288: *“Figure. 8C shows that HCV RNA abundances increased even though neither eIF4A3 mRNA or protein abundances were altered in infected cells in the presence or absence of circPSD3 (Suppl Fig. 5).”*

How many copies of circPSD3 are present in HCV infected cells in comparison to mock-infection.

Response: There are twice as many circPSD3 in infected cells than in mock-infected cells (Fig. 5C).

It is stated on line 219: *“The number of circPSD3 molecules per cell was further quantitated by Droplet Digital PCR. Results showed that approximately ten circPSD3 molecules reside per cell in mock-infected cells and twice as much in infected cells (Fig. 5C).”*

2. Does the over-expression of eIF4A3 restore the effects of circPSD3 in HCV infections?

Response: Very important point! **New Suppl. Fig. 6** shows that the effect of circPSD3 depletion on HCV RNA abundance could not be rescued by eIF4A3 over-expression in infected cells.

It is stated on line 287: *“To examine whether eIF4A3 was involved in the circPSD3-mediated modulation of HCV RNA abundances, eIF4A3 was depleted during HCV infection. Figure. 8C shows that HCV RNA abundances increased even though neither eIF4A3 mRNA or protein abundances were altered in infected cells in the presence or absence of circPSD3 (Suppl Fig. 5). This result suggests that HCV infection results in a remodeling of the NMD complex [40,42] that is less dependent on eIF4A3, or that eIF4A3 functions as an inhibitor of HCV RNA amplification. Finally, effects of ASNS RNA abundance was examined in infected cells when circPSD3 and eIF4A3 RNAs were depleted. ASNS RNA abundances diminished in circPSD3-depleted, but not in eIF4A3-depleted, infected cells (Suppl. Fig. 6A). In addition, overexpression of eIF4A3 had no effects on HCV RNA abundances (Suppl. Fig. 6B), suggesting, as mentioned above, that functional eIF4A3 complexes were not formed.”*

3. More validation is required confirm that circPSD3 diminishes NMD in uninfected and, likely, in infected cells, by sequestration of eIF4A3. How circPSD3 effect eIF4A3 at mRNA level or protein is not clear. Immunoprecipitation of eIF4A3 followed by the quantification of circPSD3 in mock and HCV-infected cells can be shown to prove the enhanced interaction between circPSD3 and eIF4A3 in HCV infected cells. Confocal analysis of colocalization between eIF4A3 and circPSD3 can also be performed.

Response: These are also great points! We examined eIF4A3 mRNA and protein abundances in infected cells (see above). Both immunoprecipitation and confocal analysis are cumbersome to perform, because of the low copy numbers of circPSD3 in uninfected and infected cells. For example, eIF4A3 is over hundred-fold more abundant in confocal analyses than circPSD3. Thus, the occasional co-localization is difficult to evaluate. Thus, besides the published HIT-Clip assays, we can not conclude with certainty that eIF4A3-circPSD3 interactions exist in infected cells. Importantly, eIF4A3 over-expression analyses indicated that circPSD3 and eIF4A3 regulate HCV RNA abundances by distinct mechanisms.

It is stated on line 271: *“It was difficult to examine direct roles circPSD3 and eIF4A3 in NMD, because of low abundance of the circRNA, and the known role for HCV core protein in the inhibition of NMD. Thus, we first examined roles for circPSD3 and eIF4A3 in NMD in uninfected cells using depletion and over-expression approaches. Using specific siRNAs, depletion of circPSD3 led to a slight reduction of NMD substrate ASNS (Fig. 8A). This suggested that circPSD3 can modulate the NMD pathway, which may contribute to its pro-viral activity during HCV infection. Interestingly, while ASNS abundance did not change when eIF4A3 was depleted, the abundance of another NMD substrate, SC35D mRNA, was significantly enhanced (Fig. 8B). Furthermore, circPSD3 depletion did not affect SC35D abundance, nor was this effect reversed in eIF4A3-depleted cells (Fig.8B). This data suggest that not all NMD substrates need the same the amount of eIF4A3 to trigger their degradation, and that eIF4A3 and circPSD3 likely modulate NMD by different mechanisms. Finally, over-expression of eIF4A3 did not affect abundances of either NMD substrate (Fig. 8A-B). It is possible that functional heteromeric eIF4A3/Y14/Magoh complexes can not be restored by simply over-expression of eIF4A3, and that circPSD3 and eIF4A3 affect NMD RNA abundances in a more substrate -specific manner.”*

**Reviewer #3:**

1. One of the novel aspects of this work is the global profiling of the circRNAs during infection. However, Fig. 1 gave very little description of these RNAs. It would be nice to have some more description of them. Are there any defining features that could be described? Do they come from RNAs that are in similar pathways? Are there pre-mRNAs upregulated or downregulated during infection?

Response: We have further explored the host genes of the differentially produced circRNAs by testing for enriched GO terms via the gprofiler2 R-package. Given the fairly small number of identified genes, this analysis yielded only the GO Cellular Component term (“nucleus”) for the host genes showing decreased circRNA abundance, whereas the genes with increased circRNA abundance were significantly enriched for the GO Molecular Function term “DNA replication origin binding”.

Line 128 states: “*To see if the host genes of these circRNAs shared common functions, we tested for enriched GO terms via the gprofiler2 R-package. However, this analysis yielded only one GO Cellular Component term (“nucleus”; p= 0.0395) for the genes showing decreased circRNA abundance, whereas genes with increased circRNA abundance were significantly enriched for the GO Molecular Function term “DNA replication origin binding” (p=0.037).”*

With regard to up- or down-regulation of the “pre-mRNAs” after viral infection, we also tried to link the transcription rate with our observations (i) by estimating pre-mRNA abundance from RNA-seq reads mapping to intronic regions only and (ii) by comparing these read counts with exonic read counts akin to how we identified alterations of circRNA abundance compared to linear transcripts. However, we did neither see any correlation between changes in a gene’s transcription rate and the occurrence of circRNAs nor were such changes in transcription exclusive for genes displaying alterations in circRNA abundance. This is not fully unexpected, as the overall transcription rate of a gene might not be predictive of such events or mask the specific increases or decreases of Pol II that could occur in case of backsplicing. Therefore, we limited our analysis to regions of 1kb flanking the affected exons, but we noticed that the resolution of our sequencing data was too limited to make any reasonable conclusions. Thus, a deeper characterization of the underlying processes might require additional data generated by Global Run On-based techniques.

2. What explains the changes in abundance of the circRNAs during infection? Is it just that their pre-mRNAs are more upregulated or downregulated?

Response: Thus, to clarify these questions to the readers, we extended our discussion to include a more detailed explanation of the possible mechanisms of HCV-induced circRNA abundance changes.

It is stated on line 308: *“It is doubtful that all identified circRNAs with altered abundances in HCV-infected cells have distinct functions, and some of them might be byproducts of altered transcription and degradation rates caused by the infection. To elaborate, as per our bioinformatics methodology, by definition all differentially produced circRNAs are exhibiting infection-induced abundance changes that cannot be explained by corresponding abundance changes of their linear counterparts. An example can be seen in the circRNA produced by TIAL1 whose abundance increases after infection, whereas its linear transcript is down-regulated. For such cases, an increased transcription rate alone does not suffice to explain the observed behavior, as one would expect both transcript types to show increases in abundance, albeit perhaps not on the same scale. Likewise, a decreased transcription rate should affect both RNAs similarly, as even a more stable circular form should not show any increases in abundance unless the backsplicing frequency has been altered as well. However, if both the transcription rate and the RNA degradation rates are simultaneously increased, the higher stability of circRNA molecules could allow them to benefit from an increased transcription rate and out-live the linear transcripts by avoiding degradation, effectively increasing their abundance while their linear counterpart’s abundance would decrease. Another possible explanation for our observations could be that HCV infection indeed alters splicing site preferences and thereby increases the likelihood for backsplicing for all identified genes, although testing this hypothesis would require a detailed analysis of the regulatory mechanisms of backsplicing before and after the viral infection. Similarly, the behavior of circRNAs exhibiting decreased abundance after infection (which we observe much more frequently as can be seen in Figure 1B) might involve alterations of the backsplicing frequency, as the linear transcripts are mostly up-regulated compared to non-infected cells. Thus, unless a circRNA-specific degradation process is induced by HCV infection for these circRNAs, alterations of backsplicing frequencies are a reasonable cause for our observations. Moreover, regardless of the involved mechanism, we are currently unable to directly predict which of the altered circRNAs are in fact functional, as circRNAs can display RNA sequences or structural motifs that are recognized by interacting RNAs or by protein molecules that modulate viral pathogenesis, such as protein kinase PKR (see below).”*

3. The authors stated that an “independent circRNA screen in HCV-infected cells identified...” It is unclear from the results section if this was an experiment that they did or were the results from published literature? Similarly, directly after they described the results as having been verified in two laboratories, but I’m not sure if they mean in the author’s labs or in published studies?

Response: The screens were done in both the Sarnow and Diez laboratories.

It is stated on line 175: “*An independent circRNA screen in both the Sarnow and Diez laboratories identified up-regulation of another circRNA, circPSD3 (Suppl. Table 1). The altered abundances of circPSD3 in liver cells during viral infection was subsequently and independently verified in both of our laboratories.”*

4. Does circPSD3 affect the production of infectious HCV virions?

Response: Yes. See **new Supp. Fig. 4**.

It is stated on line 207: “*Furthermore, circPSD3 enhanced extracellular HCV abundance, that was especially significant at a low multiplicity of infection (Supp. Fig. 4).”*

5. Does the linear RNA of PSD3 also increase during HCV infection? This could be interesting, especially if it increased prior to the production of the circRNA.

Response: Linear PSD3 RNA is often increased at later time of infection. However, circPSD3 depletion at later times of infection does not alter the abundance of linear PSD3 RNA, while HCV RNA abundance is dramatically decreased. But circPSD3 is produced at earlier times when linear PSD3 abundance has not changed.

6. The hypothesis set forth by the authors is that the circPSD3 produced during infection is proviral because it inhibits NMD, known to be antiviral. Unfortunately, it appears to be quite difficult to do the direct experiments to test them in context of infection, as the levels of the circRNA are directly related to the amount of virus replication and vice-versa. Therefore, they attempt two indirect experiments to address this point. In the first, they show during HCV infection the kinetics of circPSD3 formation and how it relates to the generation of NMD substrates. However, the circRNA appears to be made after NMD has already started, and even when present, NMD still appears to increase. Therefore, it’s not clear from this that the circRNA is slowing down NMD at all. What would happen at Day 4? At that time point, it may be that NMD starts to decrease as a result of circPSD3 upregulation at Day 3?

Response: See responses to questions # 2 and #3 from reviewer #2.

It is stated on line 267: *“While there is a correlation with inhibition of NMD and accumulation of circPSD3 in HCV-infected cells, it is noteworthy that the accumulation of circPSD3 slightly lacks behind the onset of NMD.”*

Unfortunately, infected cells start to look very sick at day 4 after infection. Thus, results from such an experiment would be hard to interpret.

7. The second set of experiments is based on the fact that others have shown the circPSD3 binds to eIF4A3, a known regulator of NMD, and so they investigate their interplay in the absence of viral infection. Specifically, the authors showed that the depletion of eIF4A3 resulted in increased NMD substrates and so reduced NMD. Based on this idea, I wonder if the authors over-expressed eIF4A3 during HCV infection, a situation which should lead to increased NMD, they could test if the circRNA has less of a regulatory effect on HCV infection? Or what about the reverse - deplete eIF4A3, reduced NMD, circRNA more important for HCV infection? I realize these experiments may be subject to some caveats, as the authors stated, but it seems like it would be important to try to get a little bit closer to proving the concept of the mechanism.

Response: Excellent points! Experiments along these lines (**new Suppl. Fig. 5, Fig. 8)** have clarified independent roles for circPSD3 and eIF4A3 in modulating HCV RNA abundance.

Its is stated on line 271: “*It was difficult to examine direct roles circPSD3 and eIF4A3 in NMD, because of low abundance of the circRNA, and the known role for HCV core protein in the inhibition of NMD. Thus, we first examined roles for circPSD3 and eIF4A3 in NMD in uninfected cells using depletion and over-expression approaches. Using specific siRNAs, depletion of circPSD3 led to a slight reduction of NMD substrate ASNS (Fig. 8A). This suggested that circPSD3 can modulate the NMD pathway, which may contribute to its pro-viral activity during HCV infection. Interestingly, while ASNS abundance did not change when eIF4A3 was depleted, the abundance of another NMD substrate, SC35D mRNA, was significantly enhanced (Fig. 8B). Furthermore, circPSD3 depletion did not affect SC35D abundance, nor was this effect reversed in eIF4A3-depleted cells (Fig.8B). This data suggest that not all NMD substrates need the same the amount of eIF4A3 to trigger their degradation, and that eIF4A3 and circPSD3 likely modulate NMD by different mechanisms. Finally, over-expression of eIF4A3 did not affect abundances of either NMD substrate (Fig. 8A-B). It is possible that functional heteromeric eIF4A3/Y14/Magoh complexes can not be restored by simply over-expression of eIF4A3, and that circPSD3 and eIF4A3 affect NMD RNA abundances in a more substrate -specific manner.*

*To examine whether eIF4A3 was involved in the circPSD3-mediated modulation of HCV RNA abundances, eIF4A3 was depleted during HCV infection. Figure. 8C shows that HCV RNA abundances increased even though neither eIF4A3 mRNA or protein abundances were altered in infected cells in the presence or absence of circPSD3 (Suppl Fig. 5). This result suggests that HCV infection results in a remodeling of the NMD complex [40,42] that is less dependent on eIF4A3, or that eIF4A3 functions as an inhibitor of HCV RNA amplification. Finally, effects of ASNS RNA abundance was examined in infected cells when circPSD3 and eIF4A3 RNAs were depleted. ASNS RNA abundances diminished in circPSD3-depleted, but not in eIF4A3-depleted, infected cells (Suppl. Fig. 6A). In addition, overexpression of eIF4A3 had no effects on HCV RNA abundances (Suppl. Fig. 6B), suggesting, as mentioned above, that functional eIF4A3 complexes were not formed. Control experiments showed that that siRNA depletion and eIF4A3 overexpression approaches were working with high efficiencies (Suppl. Fig 6C-D). These observations from both uninfected and infected cells indicate that circPSD3 and eIF4A3 affect HCV RNA abundances by distinct mechanisms, i.e. eIF4A3 by modulating specific NMD substrate abundances [39] and circPSD3 by enhancing HCV RNA abundances at a post-translational step.”*

8. Another way to get a little close to the mechanism would be to try to establish the link between circPSD3 and eiF4A3, which was not directly made in this paper. The authors showed that when eIF4A3 is knocked down, more NMD substrates are made (less NMD); in addition they showed that when you deplete circPSD3 fewer NMD substrates are made (more NMD). To show the direct link, what happens if you deplete eIF4A3 -/+ circPSD3? You might expect that if the function of circPSD is mediated by eIF4A3 sequestration to prevent NMD, if you remove eiF4A3, the circRNA should have less of an effect on this process?

 Response: see above response to comment #7.

**Part III – Minor Issues: Editorial and Data Presentation Modifications**

**Reviewer #1**:

1. The authors refer to an independent circRNA screen and altered abundance of the circRNA in response to viral infection that was verified by two independent labs. Providing references for these statements or further details would be informative.

Response: See response to point #3 made by reviewer #3 (above).

2. Although not necessary, it would be useful to larger scientific community if the authors included the data for all circRNAs altered by HCV infection as supplemental information.

Response: The data will be included in an accepted version of the manuscript.

**Reviewer #2:** (No Response)

**Reviewer #3:**

1. As you make more NMD substrates, make more circPSD3. But – does it actually inhibit?

Response: See response to your point #7. Furthermore, addition of cycloheximide did inhibit NMD as predicted (data not shown).

2. The logic of why the RNase R experiment was performed for circPSD3 was not clear. Further explanation of why this was done and why it’s important to do would be helpful.

Response: Circular RNAs do not have free ends and are thus resistant to degradation by ribo-exonucleases. Thus, most circular RNAs are resistant to exonuclease RNase R treatment.

It is stated on line 178: *“… circPSD3 was chosen for further analysis due to its highly resistance to treatment with ribonuclease RNase R, which selectively degraded single-stranded RNAs, but not circular RNAs.”*

3. In Figure 2, it might be helpful to have the name of the circRNA targeted by the siRNAs located on the bottom panel, as in siRNA: circEXOSC, below the 1, 2, and 3. This seems like it would be especially helpful for the viral infectivity panels, where as it stands now the reader would require the figure legend to know which circRNA was being targeted.

Response: Done

4. For the figure legend for Fig. 5C, it would be helpful if briefly mention how they got from copies/ul to copies/cell. It is unclear why they would change in opposite directions from the copies /ul to the copies / cell depending on the gene (e.g. GAPDH increases, but PSD3 decreases?)

Response: Done. Figure has been simplified.

5. In Figure 7, it would be helpful if the authors directly put the MOI on the figure, as opposed to having “low” or “high”. E.g MOI 0.1, MOI 1

Response: MOI has been put into the figures.

6. In Figure 7A, are the CT values for HCV RNA normalized at all or relative to a housekeeping gene? I realize that the CT value of 40 (not detected likely), could make calculating the fold change challenging.

Response: CT values have been replaced with RNA abundance in Fig. 7A.

7. Line 341 in methods – “gently” I’m assuming is meant to be generously?

Response: Corrected

8. Line 400 in methods – “HIKV” – CHIKV?

Response: Corrected
